# Ubiquitination of gasdermin D N-terminal domain directs its membrane translocation and pore formation during pyroptosis

**DOI:** 10.1038/s41419-025-07475-6

**Published:** 2025-03-17

**Authors:** Xiufeng Chu, Ting Zhang, Ihtisham Bukhari, Mei Hu, Jixuan Xu, Yamin Xing, Xinfeng Liang, Zisen Zhang, Pengyuan Zheng

**Affiliations:** 1 Department of Oncology, The Fifth Affiliated Hospital of Zhengzhou University, Zhengzhou, China; 2 Marshall B. J. Medical Research Center, The Fifth Affiliated Hospital of Zhengzhou University, Zhengzhou, China; 3 Department of Gastrointestinal & Thyroid Surgery, The Fifth Affiliated Hospital of Zhengzhou University, Zhengzhou, China

**Keywords:** Ubiquitylation, Necroptosis, Bacterial infection

## Abstract

Gasdermin D (GSDMD) is a critical pyroptosis mediator, consisting of one N-terminal pore-forming domain and one C-terminal auto-inhibitory domain. The free N-terminal domain (GD-NT), which is released through caspase-1/11 cleavage, exhibits distinct features from the full-length GSDMD (GD-FL), including oligomerization, membrane translocation, and pore-formation. However, the underlying mechanisms are not well elucidated. Here, we found that GD-NT, but not GD-FL, was massively ubiquitinated in cells. The K63-linked polyubiquitination of GD-NT at Lys236/237 (human/mouse), catalyzed by TRAF1, directly prompted its membrane translocation and pore-formation during pyroptosis. Inhibition of GD-NT ubiquitination via site-directed mutations or the UBA1 inhibitor PYR-41 suppressed cell death in several pyroptosis cell models. Additionally, applying PYR-41 in septic mice efficiently suppressed the release of IL-18 and TNFα. Thus, GD-NT ubiquitination is a key regulatory mechanism controlling its membrane localization and activation, which may provide a novel target for modulating immune activity in pyroptosis-related diseases.

## Introduction

Pyroptosis is a unique form of cell death featured by pore formation in the plasma membrane and subsequent cell swelling [1]. The release of cellular contents during pyroptosis makes it an inflammatory and immunogenic cell death (ICD). Due to these attributes, pyroptosis plays a pivotal role in the defense against pathogenic organisms as well as cancer [2, 3]. In some cases, however, it participates in the development of pathological conditions [4]. For example, pyroptosis is strongly associated with sepsis [5], Cryopyrin-Associated Periodic Syndromes (CAPS) [6], Macrophage Activation Syndrome (MAS) [7], and moderately related to type 2 diabetes [8, 9], obesity [10], myocarditis [11, 12], atherosclerotic diseases [13], gouty arthritis [14–16], and neurological diseases [17].

GSDMD is the first gasdermin that is identified as a pyroptosis mediator [18–20]. In response to canonical or noncanonical inflammasome activators, the activated inflammatory caspases (specifically, caspase-1/4/5 in humans, caspase-1/11 in mice) cleave GD-FL to release the bioactive pore-forming GD-NT fragment (the liberation of GD-NT) [21–25]. Unlike the GD-FL that exists in a monomeric state and exclusively localizes in the cytoplasm, the liberated GD-NT undergoes self-oligomerization, integrating into the plasma membrane and creating pores [24, 26, 27]. However, the mechanisms underlying the distinct biological activities between GD-FL and GD-NT are not fully understood.

Ubiquitination is a critical post-translational modification (PTM) in the regulation of inflammatory cell death by regulating the key components of NF-κB and inflammasome signaling pathways, such as NEMO, RIPK1, ASC, NLRP3, caspase-11 and IL-1β [28–34] (Extended Fig. 1). Recent studies suggest the role of ubiquitination in the stability and functions of the gasdermin family proteins. Shigella produces pathogenic ubiquitination ligase to degrade GSDMB/GSDMD and deactivate the host defensive system [35, 36]. Besides, both SYVN1-mediated ubiquitination of GSDMD and OTUD4-mediated deubiquitination of GSDME promote pyroptosis [37, 38], indicating that the host ubiquitination system also regulates gasdermin function.

**Fig. 1:**
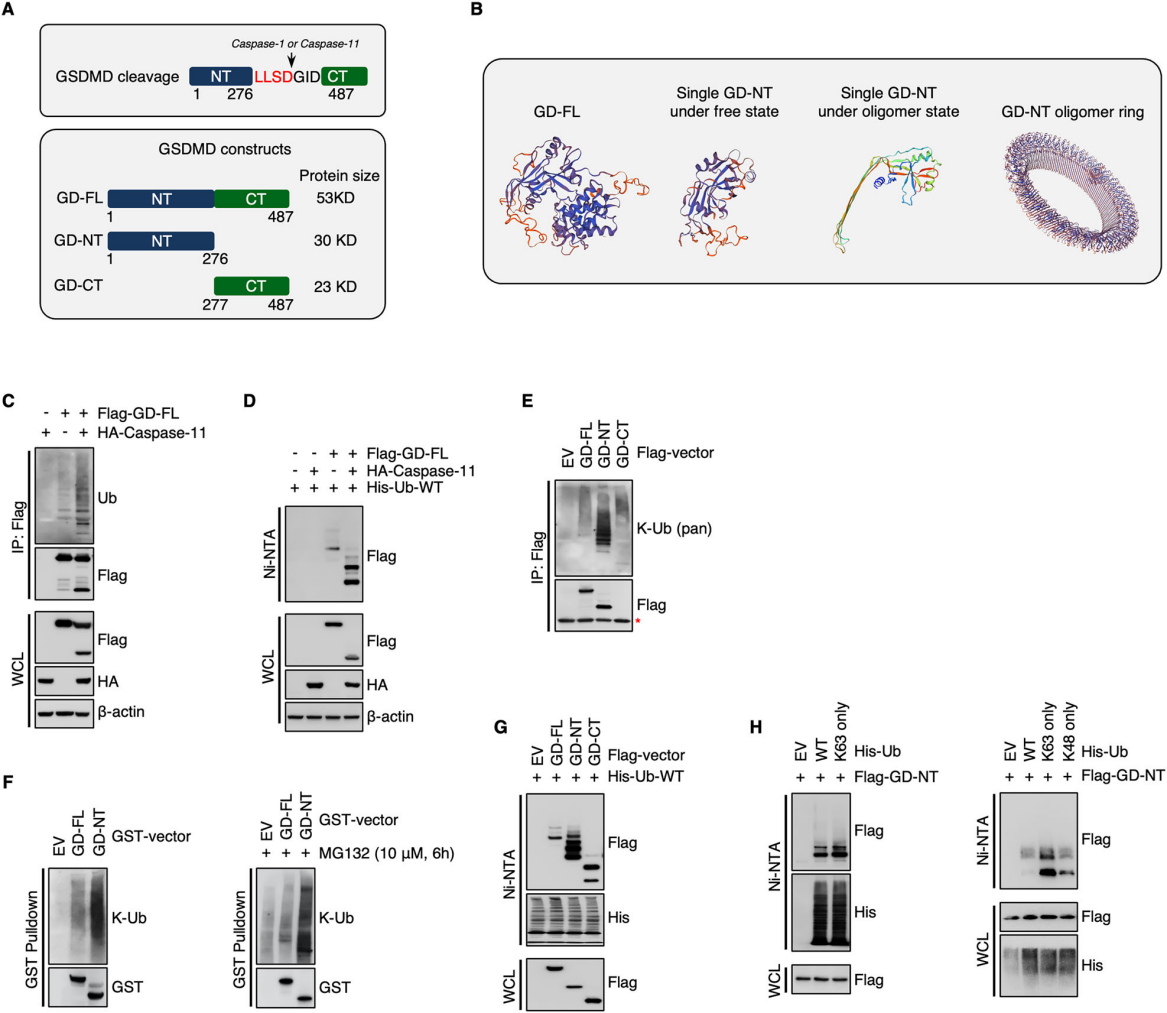
GD-NT, but not GD-FL, possesses massive K63-linked polyubiquitin. **A** Schematic illustration of mouse GSDMD cleavage, the proteases responsible for the specific cleavage, and the constructs created for the investigation of GSDMD ubiquitination. **B** Schematic illustration depicts the structure of mouse GSDMD. The structure of mouse GD-NT was downloaded from RCSB PDB (PDB code: 6N9N), and PyMOL was used to generate a structure model of GD-FL, GD-NT. **C** 293 T cells were transfected with Flag-GD-FL and/or caspase-11. 24 hours (h) later, the cells were harvested and subjected to Flag-IP assay. The precipitants and WCL (whole cell lysate) were immunoblotted with the indicated antibodies. **D** His-Ub was transfected with Flag-GD-FL and/or caspase-11 in 293 T cells. 24 hours later, the cells were harvested and subjected to Ni-NTA Pulldown assay. The precipitants and WCL were immunoblotted with the indicated antibodies. **E** Similar to (**B**), except that 293 T cells were transfected with Flag-GD-FL, NT, or CT, respectively. The mark “*” indicated that the band of GD-CT overlapped with the unspecific band from the anti-Flag primary antibody. **F** 293 T cells were transfected with GST-GD-FL or NT and treated with or without MG132 (10 μM, 6 h). 24 hours later, the cells were harvested and subjected to GST Pulldown assay. The precipitants and WCL were immunoblotted with the indicated antibodies. **G** Similar to (**C**), except that His-Ub was transfected with Flag-GD-FL, NT, or CT, respectively. **H** His-Ub-WT, His-Ub-K63 only, or His-Ub-K48 only was transfected with Flag-GD-NT in 293 T cells. 24 hours later, the cells were harvested and subjected to Ni-NTA Pulldown assay. The precipitants and WCL were immunoblotted with the indicated antibodies. In the construct His-Ub-K63 only and His-Ub-K48 only, all other lysines of ubiquitin were substituted with arginine except for Lys63 and Lys48, respectively. In **C**, **E** and **F**, to keep the integrity of the ubiquitin chain attached to substrate proteins, NEM (10 mM) was added to inhibit the activity of cysteine peptidases. To prevent the influence of GSDMD interactome, protein samples for IP were denatured via heating at 100 °C for 5 minutes to break protein-protein interaction.

Here, we report that the ubiquitination at Lys237 of GD-NT (but not GD-FL) is a prerequisite mechanism of GSDMD activation. This process enables GD-NT to translocate and form pores in the plasma membrane, thereby causing pyroptotic cell death. Therefore, we identified a novel molecular mechanism of the liberated GD-NT acquiring pyroptotic ability.

## Results

### GD-NT, but not GD-FL, possesses K63-linked polyubiquitination

To investigate the ubiquitination of GSDMD, the vectors expressing GD-FL, GD-NT, GD-CT, or caspase-11 were constructed (Fig. 1A, B). We transfected Flag-GD-FL/caspase-11 into HEK293 cells and performed IP & immunoblot assay. Interestingly, the result showed that GSDMD was slightly ubiquitinated when it was introduced into the cells alone. However, the ubiquitination level of GSDMD was significantly increased when it was co-transfected with caspase-11 (Fig. 1C). To further ensure it, we adopted a more rigorous ubiquitination detection method, in which we denatured the cellular proteins using guanidine and subjected to NI/NTA pulldown (Fig. 1D). Immunoblot (IB) analysis of the precipitants revealed the same ubiquitination pattern as shown in Fig. 1C. We supposed that the alteration of ubiquitination may take place during GSDMD processing by caspase-11. To test this hypothesis, we independently transfected Flag-GD-FL, Flag-GD-NT, or Flag-GD-CT into HEK293 cells. 18 hours later, the cells were subjected to IP assay. IB analysis of the precipitants revealed that GD-NT had a distinct ubiquitination status from GD-FL (Fig. 1E). Later on, we performed GST-pulldown and NI/NTA pulldown assay under denatured conditions. Consistently, these assays also indicated that GD-NT, but not GD-FL or GD-CT, was massively ubiquitinated (Fig. 1F, G).

To further identify the conjugation form of ubiquitin on GD-NT, we transfected Flag-GD-NT along with either Ub-WT or Ub-K63 only into HEK293 cells and then performed an NI/NTA pulldown assay. The result showed that GD-NT was mainly conjugated with K63-linked polyubiquitin chains (Fig. 1H). Thus, our data suggest that GD-NT, but not GD-FL, is massively modified with K63-ubiquitination.

### The K63-linked polyubiquitination of GD-NT is regulated by TRAF1-OTUB1 axis

**Fig. 2:**
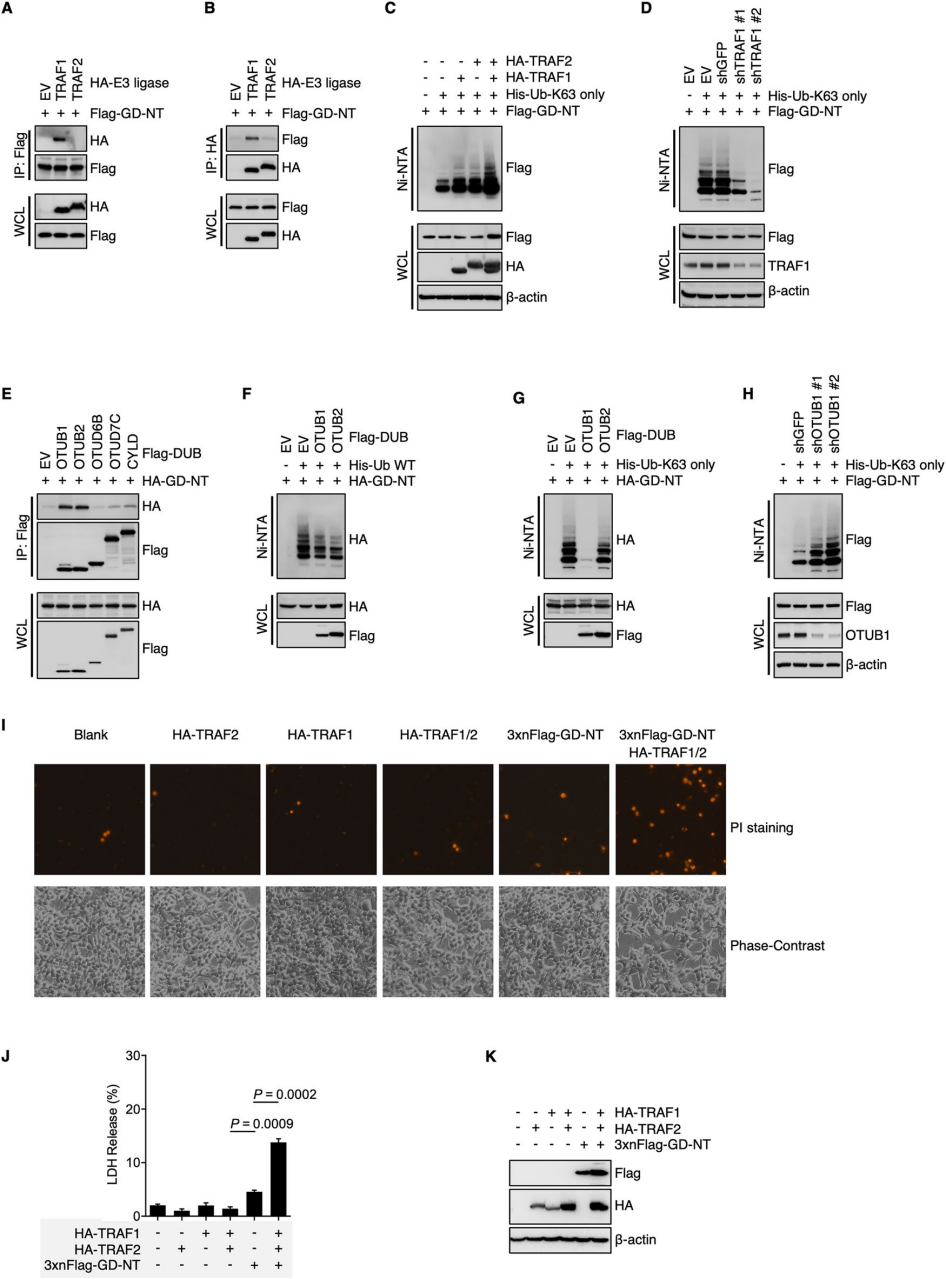
The K63-linked polyubiquitin of GD-NT is regulated by TRAF1-OTUB1 axis. **A**, **B** Interaction between GD-NT and E3 ligases. HEK293T cells were transfected with Flag-GD-NT and different HA-E3. 24 hours later, the cells were harvested for Flag-IP assay (**A**) and HA-IP assay (**B**). The preciptants were subjected to immunoblot with the indicated antibodies. **C**, **D** Deubiquitylation of GD-NT by DUBs. HEK293T cells were transfected with Flag-GD-NT, His-Ub-K63 only, and HA-TRAF/2 (**C**) or shTRAF1 (**D**). 24 hours later, the cells were harvested for Ni-NTA pull-down assays. The precipitants and WCL were immunoblotted with the indicated antibodies. **E** GD-NT interaction with DUBs. HEK293T cells were transfected with HA-GD-NT and different Flag-DUB. 24 hours later, the cells were harvested for IP assay. The preciptants were subjected to immunoblot with the indicated antibodies. **F** Deubiquitylation of GD-NT by DUBs. HEK293T cells were transfected with HA-GD-NT, His-Ub-WT, and Flag-OTUB1 or Flag-OTUB2. 24 hours later, the cells were harvested for Ni-NTA pull-down assays. The precipitants and WCL were immunoblotted with the indicated antibodies. **G** HEK293T cells were transfected with HA-GD-NT, His-Ub-K63 only, and Flag-OTUB1 or Flag-OTUB2. 24 hours later, the cells were harvested for Ni-NTA pull-down assays. The precipitants and WCL were immunoblotted with the indicated antibodies. **H** Similar to (**G**), except that shOTUB1 was used to knock down the expression of OTUB1. **I** TRAF1 and TRAF2 enhance the cytolytic activity of GD-NT. HEK293 cells were transfected with 3xFlag-GD-NT with or without HA-TRAF1 and HA-TRAF2. 48 hours later, morphological changes were observed using phase-contrast imaging (lower panel). Dying cells were stained with PI and photographed using fluorescent microscope (upper panel). **J** Similar to (**I**), except that the cells were subjected to LDH-based Cytotoxicity Assay. **K** Similar to (**I**), except that the WCL were immunoblotted with the indicated antibodies.

Ubiquitination is a dynamic and reversible process in which E3 ligases and deubiquitinases (DUB) catalyze the conjugation and removal of ubiquitin on substrate proteins, respectively [39, 40]. In addition, the E3 ligase-DUB axis exhibits its specificity by determining the target proteins and their ubiquitination forms. To identify the E3 ligase responsible for the K63-linked ubiquitination of GD-NT, we co-transfected Flag-GD-NT with various E3 ligases into HEK293 cells and conducted an IP assay. IB analysis of the precipitants indicated that Flag-GD-NT interacted with TRAF1 (Fig. 2A, B). To assess the influence of TRAF1 on GD-NT ubiquitination, we transfected either TRAF1 or shTRAF1 together with Ub-K63 only and Flag-GD-NT into HEK293 cells, followed by an NI/NTA pulldown assay. We found that TRAF1 significantly enhanced the K63-linked ubiquitination of GD-NT, whereas the ubiquitination level was downregulated by shTRAF1 (Fig. 2C, D). Interestingly, although the interaction of GD-NT with TRAF2 was not observed in our earlier experiments (Fig. 2A, B), TRAF2 still enhanced the K63-linked ubiquitination of GD-NT (the last two lanes in Fig. 2C). This phenomenon, we believe, may be attributed to the formation of a heterodimer between TRAF1 and TRAF2 [41], or to the involvement of another factor that has yet to be identified.

Next, to identify the DUB responsible for the K63-linked ubiquitination of GD-NT, we transfected Flag-GD-NT along with different DUBs into HEK293 cells and performed an IP assay. The result showed that Flag-GD-NT interacted with both OTUB1 and OTUB2 (Fig. 2E). Subsequently, either Ub-WT or Ub-K63 only was co-transfected with HA-GD-NT and/or OTUB1/2, followed by NI/NTA pulldown. IB analysis of the precipitants showed that, although both OTUB1 and OTUB2 affected the ubiquitination of GD-NT, only OTUB1 significantly reduced the K63-linked ubiquitination of GD-NT (Fig. 2F, G). Consistently, shRNA-mediated silencing of OTUB1 significantly increased the K63-linked ubiquitination of GD-NT (Fig. 2H). Moreover, we found that 3 × Flag-GD-NT, which loses its pore-forming activity due to the addition of triple Flag-tag on its N-terminal [3], regained the ability to mediate pyroptosis with the assistance of TRAF1 and TRAF2 (Fig. 2I–K). Therefore, our findings suggest that the K63-linked ubiquitination of GD-NT is regulated by the TRAF1-OTUB1 axis, which in turn influences the pyroptotic activity of GD-NT.

### GD-NT possesses the K63-linked polyubiquitination through its Lys237

**Fig. 3:**
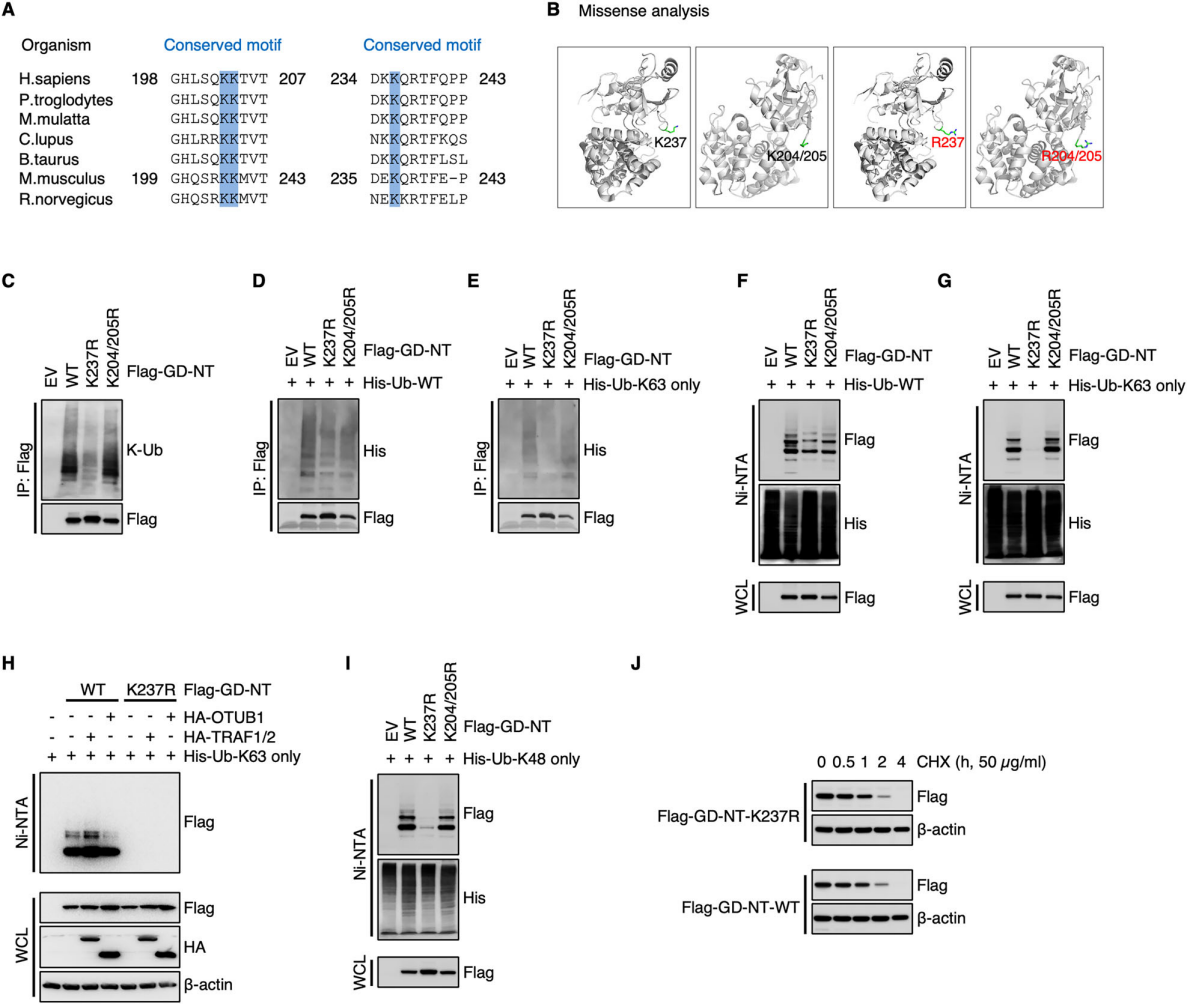
The amino acid Lys237 of GD-NT possesses K63-type ubiquitination. **A** Lys237 of GD-NT is evolutionarily conserved among various organisms, including H. sapiens, P. troglodytes, M. mulatta, C. lupus, B. taurus, M. musculus, and R. norvergicus. **B** Structural damage analysis of GSDMD site mutation. Structural damage was assessed using the online tool Missense3D (http://www.sbg.bio.ic.ac.uk/missense3d). No structural damage was detected during the creation of GD-NT mutants via site-directed mutagenesis. **C** HEK293 cells were transfected with Flag-GD-NT, K237R, K204/205R. 24 hours later, the cells were harvested for Flag-IP assay. The precipitants and WCL were immunoblotted with the indicated antibodies. **D** His-Ub-WT was transfected with Flag-GD-NT, K237R, K204/205R into HEK293 cells. 24 hours later, the cells were harvested for Flag-IP assay. The precipitants and WCL were immunoblotted with the indicated antibodies. **E** Similar to (**D**), except that His-Ub-K63 only was used instead of His-Ub-WT. **F** His-Ub-WT was transfected with Flag-GD-NT, K237R, K204/205R into HEK293 cells. 24 hours later, the cells were harvested for Ni/NTA pulldown assay. The precipitants and WCL were immunoblotted with the indicated antibodies. **G** Similar to (**F**), except that His-Ub-K63 only was used instead of His-Ub-WT. **H** HEK293T cells were transfected with Flag-GD-NT and HA-TRAF1/2 or HA-OTUB1. 16 hours later, the cells were harvested for Ni-NTA pull-down assays. The precipitants and WCL were immunoblotted with the indicated antibodies. **I** Similar to (**F**), except that His-Ub-K48 only was used instead of His-Ub-WT. **J** HEK293 cells were transfected with Flag-GD-NT-WT or K237R. 12 hours later, the cells were treated with cycloheximide (CHX, 50 μg/mL) and then harvested at different time points for Flag-IP assay. The WCL were immunoblotted with the indicated antibodies. In **C**, **D**, and **E**, to keep the integrity of the ubiquitin chain attached to substrate proteins, NEM (10 mM) was added to inhibit the activity of cysteine peptidases. To prevent the influence of GSDMD interactome, protein samples for IP were denatured via heating at 100 °C for 5 minutes to break protein-protein interactions.

To identify the ubiquitin conjugation sites on GD-NT, we searched the online PhosphoSitePlus® database (PSP, https://www.phosphosite.org), which provides comprehensive information for the study of ubiquitination as well as other post-translational modifications (PTMs) [42]. Based on Low ThroughPut and High ThroughPut datasets obtained from the PSP database [38, 43], several amino acids were considered as the potential ubiquitination sites, including Lys204, Lys205, and Lys237 in mouse GSDMD (refer to Lys203, Lys204, and Lys236 in humans). Sequence alignment of GSDMD among different species revealed an evolutionary conservation of these lysine residues (Fig. 3A).

To assess the ubiquitination of Lys204, Lys205, and Lys237 of GD-NT (but not GD-FL), we created the non-ubiquitinatable mutants GD-NT-K237R and GD-NT-K204/205R. First, we performed Missense3D analysis and found no evidence of artificial structural damage resulting from these “K to R” substitutions, indicating that these constructs could be used for subsequent ubiquitination studies (Fig. 3B). Then, we transfected HEK293 cells with GD-NT-WT, GD-NT-K237R or GD-NT-K204/205R and conducted IP assay. The results showed that K237R, but not K204/205R, significantly impaired the K63-linked polyubiquitination of GD-NT (Fig. 3C–E). This difference was even more obvious in the subsequent NI-NTA pulldown assay (Fig. 3F, G). Consistently, we found that the TRAF1-OTUB1 axis affected GD-NT-WT, but not the mutant GD-NT-K237R, for K63-linked polyubiquitination (Fig. 3H). These findings indicate that K63-linked polyubiquitination of GD-NT primarily occurs at Lys237, and this event is regulated by the TRAF1-OTUB1 axis.

Intriguingly, we observed that GD-NT-K237 also possessed K48-linked polyubiquitination, which is primarily associated with proteasomal degradation (Fig. 3I). However, this modification did not affect GD-NT stability (Fig. 3J), suggesting that the K48-linked polyubiquitination on GD-NT-K237 represents a non-functional modification.

### The K63-linked polyubiquitination controls GD-NT pyroptotic activity

**Fig. 4:**
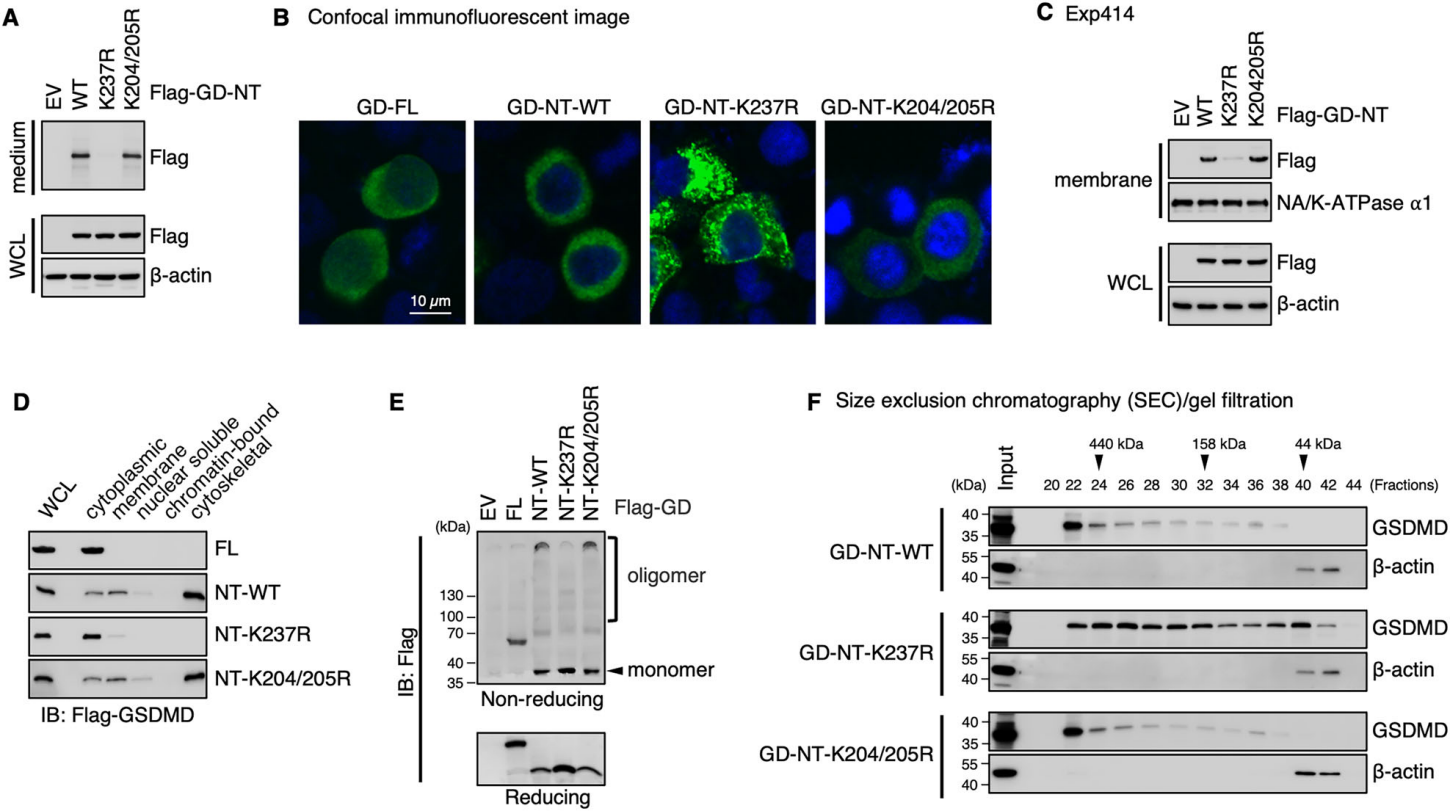
Ubiquitination on Lys237 of GD-NT affects its localization and oligomerization. **A** Detection of GD-NT in culture medium. HEK293 cells were transfected with the indicated constructs. 16 hours later, the culture medium and WCL were harvested and immunoblotted with the indicated antibodies. **B** Distribution pattern of GD-NT-WT or its mutants. HeLa cells were transfected with the indicated constructs. Representative confocal microscopy images showing distribution of ectopic Flag-GD-FL, GD-NT-WT, GD-NT-K237R, or GD-NT-K204/205R (green) co-stained with DAPI (blue). **C**, **D** Subcellular localization of GD-NT-WT or its mutants. HEK293 cells were transfected with the indicated constructs. 16 hours later, cells were harvested and subjected to sample preparation using a Thermo Fisher Subcellular Fractionation Kit. Membrane fractions (**C**), and other cellular fractions (**D**), were immunoblotted with the indicated antibodies. WCL was included as an input control. **E** Oligomerization of GD-NT. HEK293 cells were transfected with the indicated constructs. 16 hours later, cells were harvested and subjected to IB analysis with the indicated antibodies under reducing or non-reducing conditions. **F** Fractionation via SEC/gel filtration. HEK293 cells were transfected with the indicated constructs. 16 hours later, cells were harvested for fractionation with SEC/gel filtration. Eluent protein fractions were immunoblotted with the indicated antibodies. Input represents 10% of proteins used for SEC/gel filtration. Data are representative of at least two independent experiments.

As a specific pattern of PTMs, the K63-linked polyubiquitination generally controls various properties of the proteins, including protein-protein interaction, translocation, and activation. To assess whether the K63-linked polyubiquitination of Lys237 influences GD-NT pore-forming and cytolytic activity, we transfected HEK293 cells with GD-NT-WT, GD-NT-K237R or GD-NT-K204/205R. 24 hours later, culture medium and whole cell lysate were harvested for immunoblot analysis, respectively. Interestingly, GD-NT-K237R was not detected in the culture medium, suggesting that this GD-NT mutant can not permeabilize the plasma membrane and translocate into the culture medium (Fig. 4A).

To determine how the K63-linked polyubiquitination of Lys237 affects GD-NT cytolytic activity, we further investigated the subcellular localization and oligomerization of GD-NT-K237R using various strategies [26]. First, we transfected HeLa cells with GD-FL, GD-NT-WT, GD-NT-K237R, or GD-NT-K204/205R and then performed confocal immunofluorescent microscopy to visualize the localization of these constructs. Our result showed that GD-NT-K237R clustered near the plasma membrane, which was different from GD-NT’s even distribution along the plasma membrane (Fig. 4B).

To precisely define GD-NT-K237R subcellular localization, HEK293 cells were transfected with these constructs and then fractionated into five compartments (soluble cytoplasmic, membrane, soluble nuclear, chromatin-bound nuclear, and insoluble cytoskeletal content) for immunoblot analysis. These results showed that GD-NT-K237R was less abundant in the membrane and insoluble cytoskeletal fractions in comparison with GD-NT-WT (Fig. 4C, D). Next, to assess the oligomer formation of GD-NT-K237R, HEK293 cells were transfected with these constructs and subsequently subjected to immunoblot under non-reducing conditions. We found that GD-NT-K237R failed to form the oligomers (Fig. 4E). In addition, these transfected cells were fractionated through size exclusion chromatography (SEC)/gel filtration under native conditions and then subjected to immunoblot under reducing conditions. The result revealed that GD-NT-K237R exhibited a diffuse distribution pattern, whereas GD-NT-WT formed a single huge oligomer (≥440 kDa) (Fig. 4F). Collectively, these findings suggest that GD-NT loses pyroptotic activity when it is modified with K63-linked polyubiquitin chains at Lys237.

### GD-NT-K237R fails to mediate pyroptosis in vitro and in vivo

**Fig. 5:**
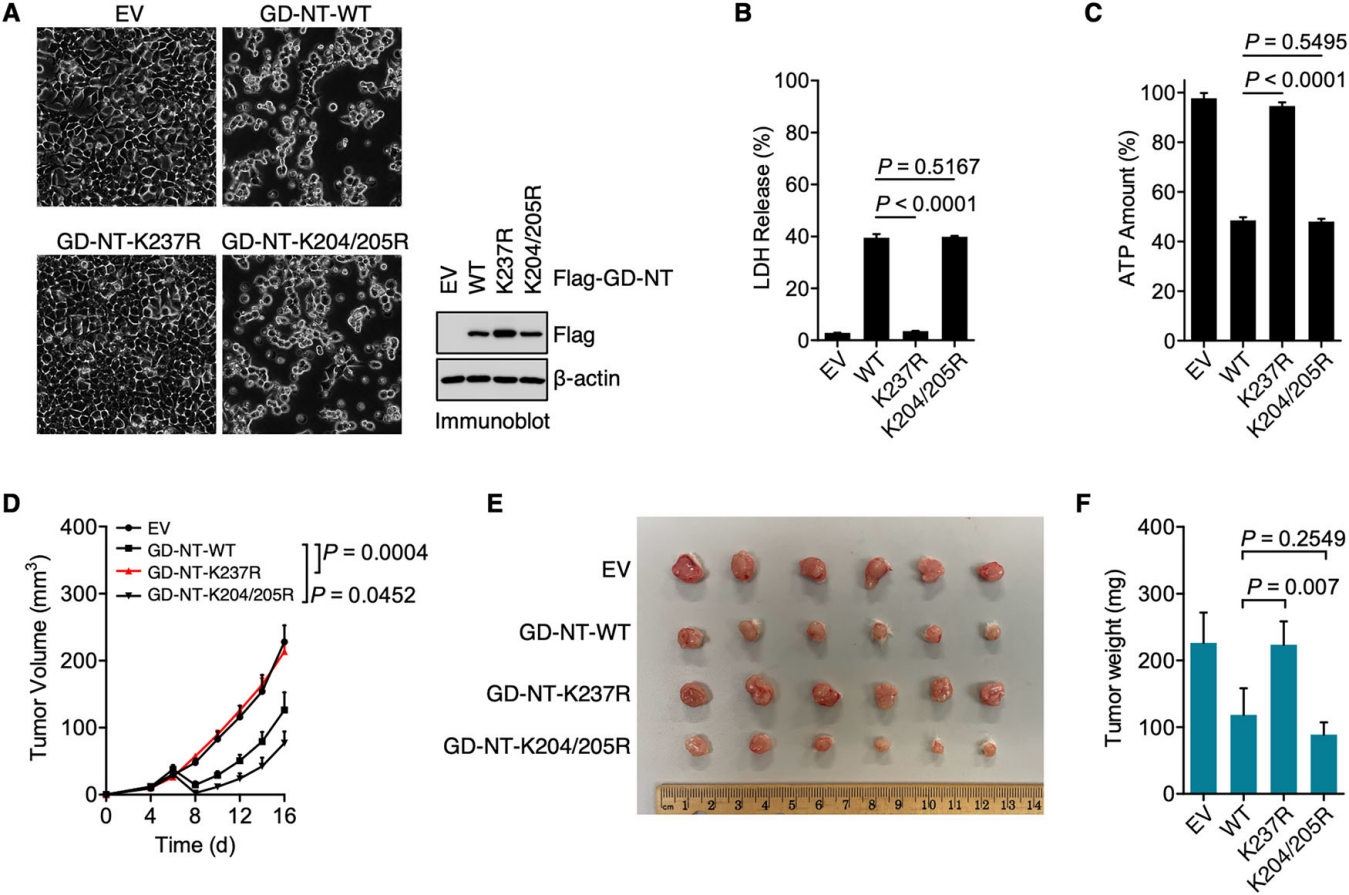
Ubiquitinated GD-NT loses the pyroptosis-mediating ability in vivo and in vitro. **A** HEK293 cells were transfected with Flag-GD-NT-WT or its mutants. Phase-contrast images were taken 24 hours after transfection (left panel). In addition, the cells were harvested for IB analysis 16 hours after transfection (right panel). **B** Similar to (**A**), except that the cells were harvested for LDH-based Cytotoxicity Assay 24 hours after transfection. **C** Similar to (**A**), except that the cells were harvested for ATP-based Cell Viability Assay 24 hours after transfection. **D**–**F** HeLa xenograft in NSG mice. HeLa cells expressing Dox-inducible GD-NT-WT or its mutants (0.5 × 10^6^ cells per mouse) were subcutaneously implanted into the right flank of NSG mice (*n* = 6 mice per group). Dox (50 mg/kg, i.p.) was administrated on day 6, 8, 10, 12, 14 and 16. Tumor growth was recorded every other day. Shown are tumor growth (**D**), tumor image (**E**), and tumor weight (**F**). In **B**, **C**, **D**, and **F**, error bars represent a variation range of duplicated experiments. In **B**, **C**, and **F**, differences among groups were analyzed by two-tailed Student’s *t*-test (means ± s.e.m). In **D**, the areas under the growth curves were compared by a two-tailed Student’s *t*-test (means ± s.e.m). Data are representative of at least two independent experiments.

To directly evaluate the influence of ubiquitination on GD-NT-mediated pyroptosis, HEK293 cells were transfected with GD-NT-WT, GD-NT-K237R, or GD-NT-K204/205R and then subjected to cell death/survival assessment. Phase-contrast imaging indicated that, unlike GD-NT-WT and GD-NT-K204/205R that exhibited cellular toxicity, the mutant GD-NT-K237R caused no obvious morphological changes (Fig. 5A). Consistently, subsequent LDH-based cell death assay and ATP-based cell viability assay suggested that GD-NT-K237R was incapable of mediating pyroptosis (Fig. 5B, C). To further investigate the pyroptotic activity of GD-NT-K237R, we employed a xenograft model in NSG mice using HeLa cells that have doxycycline (Dox)-inducible expression of GD-NT-WT, GD-NT-K237R or GD-NT-K204/205R. Dox was administered via intraperitoneal injection to induce the expression of GD-NT. Similar to in vitro findings, the xenografts expressing GD-NT-WT had a slower growth rate than those expressing the empty vector. In contrast, GD-NT-K237R did not efficiently suppress xenograft growth (Fig. 5D–F). These data suggest that the K63-linked polyubiquitination on Lys237 prevents GD-NT from mediating pyroptosis.

### UBA1 inhibitor PYR-41 suppresses GD-NT-mediated pyroptosis in vitro and in vivo

As K63-linked ubiquitination plays a positive role in GD-NT pyroptotic activity, we sought to evaluate the effects of ubiquitination-targeting small molecules on GD-NT-mediated pyroptosis. Due to the absence of selective molecules targeting TRAF1 or OTUB1, we instead evaluated the universal ubiquitination modulators, including UBA1 inhibitors PYR-41 and MLN7243, NAE inhibitor MLN4924, SAE inhibitor TAK-981 and the proteasome inhibitor Bortezomib.

**Fig. 6:**
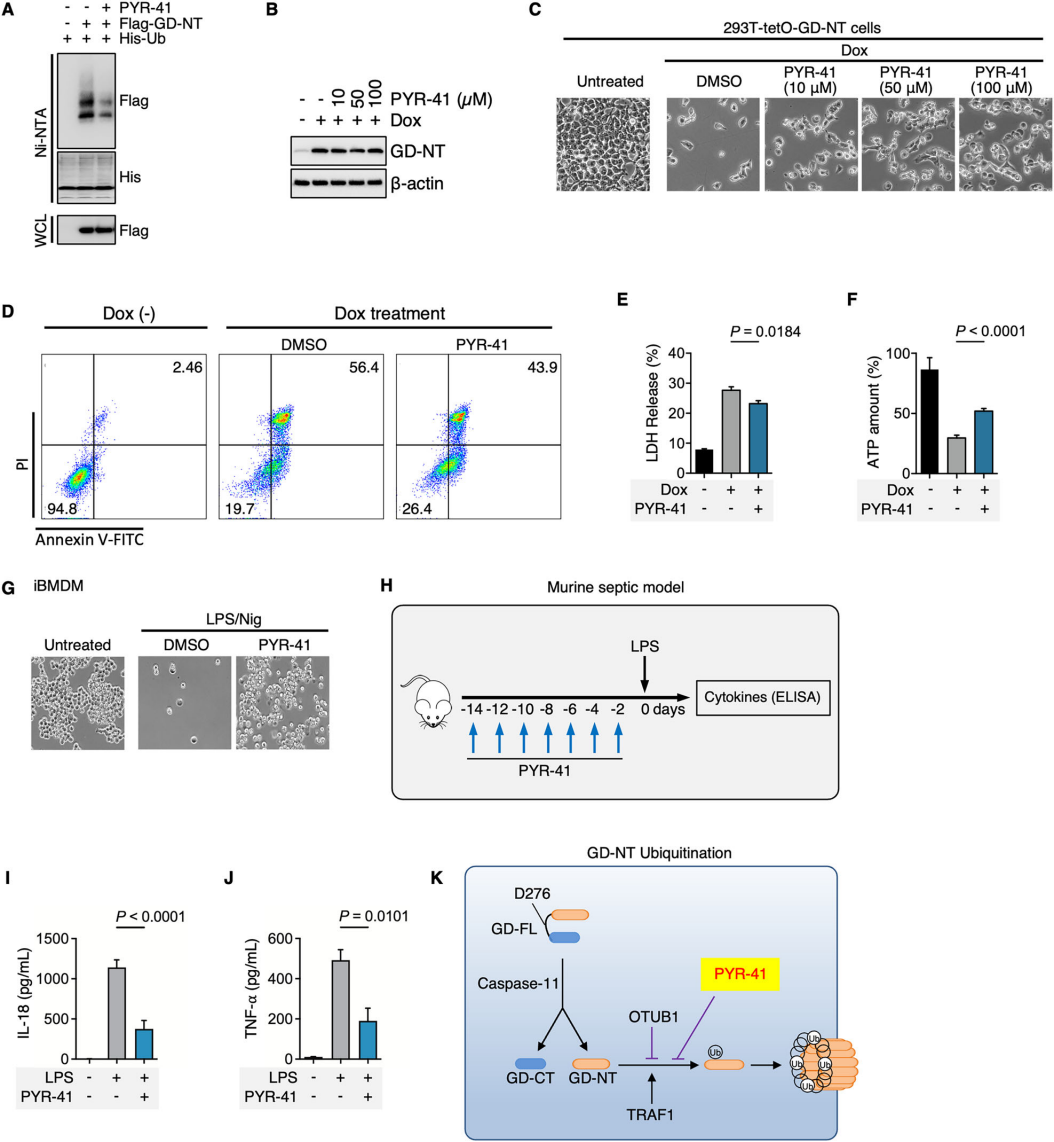
PYR-41 suppressed GD-NT ubiquitination and reduced GD-NT-mediated pyroptosis in vivo and in vitro. **A** 293-tetO-His-Ub cells were used for ubiquitination detection. Dox (2 µg/mL) was added to induce the expression of His-Ub. 24 hours after Dox treatment, the cells were transfected with Flag-GD-NT and simultaneously treated with PYR-41 (50 µM). 24 hours after transfection, the cells were harvested for Ni-NTA Pulldown. The precipitants and WCL were immunoblotted with the indicated antibodies. **B** 293-tetO-GD-NT cells were treated with Dox (2 µg/mL) to induce GD-NT expression. Different concentrations of PYR-41 were added at the same time as Dox. 6 hours after Dox/PYR-41, and the cells were harvested for immunoblot with the indicated antibodies. **C** Similar to (**B**), except that phase-contrast images were taken 16 hours after Dox/PYR-41. The cells floating in the culture medium were removed, and the adherent cells (considered as living cells) were subjected to phase-contrast imaging. **D** 293-tetO-GD-NT cells were treated with Dox (2 µg/mL) to induce GD-NT expression. PYR-41 (50 µM) was added at the same time as Dox. 12 hours after Dox/PYR-41, the cells were harvested for flow cytometry and Annexin V/PI staining kit. **E** Similar to (**D**), except that the cells were harvested 12 hours post-Dox and subjected to LDH-based Cytotoxicity Assay. **F** Similar to (**D**), except that the cells were harvested 12 hours post-Dox and subjected to ATP-based Cell Viability Assay. **G** Phase-contrast images of iBMDM cells. The cells were treated with LPS (1 μg/mL) and (PYR-41 50 μM)) for 10 hours before stimulation with nigericin for 30 minutes. The cells floating in the culture medium were removed, and the adherent cells (considered as living cells) were subjected to phase-contrast imaging. **H** Murine septic model: LPS, with or without PYR-41, was administered according to the formula. **I**–**J** Cytokine release in septic mice. LPS (5 mg/kg), with or without PYR-41 (10 mg/kg), was intraperitoneally injected into C57BL/6 naïve mice (*n* = 6 mice per group). 8 hours after LPS, the sera were harvested for ELISA assay to detect IL-18 (**I**) and TNFα (**J**) levels. **K** Diagrammatic illustration: The ubiquitination of GD-NT mediated by TRAF1/2 and OTUB2 enables GD-NT to polymerize and form pores in the cell membrane. The UBA1 inhibitor PYR-41 can significantly block GD-NT-mediated cell apoptosis. In **E**, **F**, **I**, and **J**, differences among groups were analyzed by two-tailed Student’s *t*-test (means ± s.e.m). Error bars represent the variation range of duplicated experiments. Data are representative of at least two independent experiments.

First, to test the influence of PYR-41 on GD-NT ubiquitination status, HEK293 cells were transfected with His-Ub and Flag-GD-NT and then treated with PYR-41. Ni/NTA pulldown assay indicated that PYR-41 significantly reduced the ubiquitination of GD-NT (Fig. 6A).

Next, to evaluate the effects of PYR-41 on GD-NT-mediated pyroptosis, we established a pyroptosis model cell line, “293-tetO-GD-NT”, in which the transgene GD-NT is expressed in response to Dox. Immunoblot assay revealed a peak expression of GD-NT at 4^th^ hour post-Dox. Fluorescent imaging indicated that more than 95% of cells underwent pyroptosis at 24^th^ hour of the Dox treatment (Extended Fig. 2). We then used 293-tetO-GD-NT cells to assess the effects of PYR-41 on GD-NT-mediated pyroptosis via different methods, including Phase-contrast imaging, Flow cytometry, LDH-based cell death assay, and ATP-based cell viability assay. Notably, PYR-41 treatment significantly reduced pyroptosis in 293-tetO-GD-NT cells (Fig. 6B–F).

Sepsis is the leading cause of death in intensive care units (ICUs) across the globe [44]. It is characterized by sustained excessive inflammation and immune suppression as well as organ dysfunction. Failure of more than a hundred clinical trials for finding a possible cure is attributed to the complexity of mediators and pathways involved in sepsis. Accumulating evidence suggests that GD-NT-mediated pyroptosis of macrophages is the main pathological basis of sepsis [5, 45], and the potential therapeutic targets in pyroptosis may provide future direction for sepsis treatment. In this study, we investigated the effects of PYR-41 in LPS/Nigericin-induced pyroptosis of immortalized bone marrow-derived macrophages (iBMDM) and LPS-induced septic mice. Surprisingly, we found that PYR-41 reduced iBMDM pyroptosis as well as the release of IL-18 and TNFα in septic mice (Fig. 6G–J). Thus, we concluded that inhibition of GD-NT ubiquitination suppresses its mediated pyroptosis and reduces the severity of septic condition in mice (Fig. 6K).

On the other hand, in the context of cancer, GD-NT-mediated pyroptosis of tumor cells releases numerous immunogenic products and may enhance anti-tumor immunity [1, 4, 46, 47]. Pharmaceutical modulation of GD-NT ubiquitination in cancer requires further investigations.

## Discussion

Although originating from the same amino acid sequence, GD-FL and the liberated GD-NT are two proteins that exhibit significant functional differences. First, GD-FL solely stays in the cytoplasm, while GD-NT spreads in both the cytoplasm and plasma membrane. Second, GD-FL is generally found in a monomeric state, while GD-NT as an oligomeric state. Previously, we reported that GD-FL and GD-NT have different interactomes [26]. An increasing number of studies suggest that the distinct biological features can be attributed to Post-Translational Modifications (PTMs). For example, the intermediate of the tricarboxylic acid cycle, fumarate, and its dimethyl form DMF react with the cysteine residue of GD-FL to form succinates, thus preventing GD-FL from interacting with caspase-11 and the subsequent cleavage [48]. With the help of palmitoyl acyltransferases ZDHHC5 and ZDHHC9, GD-FL undergoes palmitoylation at Cys191/192 (human/mouse). This modification does not affect the cleavage of GD-FL by inflammatory caspases, but it improves the translocation of GD-NT to the plasma membrane and form pores in a much more efficient way [49]. Moreover, we previously found that AMPK interacts with GD-NT but not GD-FL. AMPK phosphorylates the Ser46 residue of GD-NT to suppress its translocation and pore-forming activity [26] (Extended Fig. 3). These modifications affect the functions of GD-FL and GD-NT by regulating their structures, localization, stability, and trafficking.

Ubiquitination of gasdermin has drawn our attention due to the works of Vishva and Alto in 2021 [35, 36]. They found that the ubiquitin ligase IpaH7.8 secreted by intracellular Shigella causes the ubiquitination and subsequent degradation of GSDMD and GSDMB, thereby disabling the host defensive system of pyroptosis (Extended Fig. 4). Given that mammalian cells hold a more complex ubiquitination system, we supposed that the processing and functioning of gasdermin proteins are tightly regulated by the precise conjugation of ubiquitin in host cells. In the current study, we found that only GD-NT, but not GD-FL, underwent K63-type polyubiquitinationon Lys237, which directed its translocation and oligomerization. The TRAF1-OTUB1 axis was responsible for the ubiquitination and de-ubiquitination of GD-NT-K237. Additionally, we noticed that the ubiquitination on Lys237 did not affect the cleavage of GD-FL by caspase-11 and the production of GD-NT. Thus, we concluded that GD-NT possesses a unique ubiquitination status that differs significantly from GD-FL, and the ubiquitination on Lys237 is a post-cleavage event that helps GD-NT translocation, forming pores in the plasma membrane and therefore promoting pyroptosis. Similarly, the UBA1 (E1) inhibitor PYR-41 significantly suppressed GD-NT-mediated pyroptosis in 293-tetO-GD-NT cells, iBMDM, and septic mice.

Thus, our study elucidates the unique role of ubiquitination in the regulation of GD-NT-induced pyroptosis. In addition, we believe that manipulation of PTMs of GD-FL or GD-NT by small molecules, such as PYR-41, DMF, palmostatin B (PMB), metformin, and disulfiram [50], is potentially paving a therapeutic path in pyroptosis-related diseases. Intriguingly, some of these chemicals have exhibited promising outcomes in the animal models of septic shock [51] (Extended Fig. 5).

## Materials and methods

### Data reporting

No statistical methods were used to predetermine the sample size. The samples were not randomized. The investigators were not blinded to allocation during the experiments and the evaluation of the results.

### Cell lines and cell culture conditions

HEK293, HeLa, and iBMDM cells were purchased from ATCC and were maintained in Dulbecco’s Modified Eagle’s Medium (DMEM) supplemented with 10% heat-inactivated fetal bovine serum (FBS), 100 U/mL penicillin, and 100 μg/mL streptomycin. All cells were tested for mycoplasma by PCR and also authenticated by morphology.

### Plasmids

pDB-His-MBP-mGSDMD (Addgene, 123365) was gifted by Hao Wu. pTRIPZ-shNS (Addgene, 127696) was received as a gift from Sandra Demaria. The vectors expressing GD-NT, human TRAFs, and human DUBs were generated by the standard PCR cloning strategy. Truncation mutation or point mutation plasmids were generated using the QuickChange Primer Design Program and mutagenesis kit (Agilent Technologies). All plasmids were verified by DNA sequencing and IB analysis.

### Reagents and antibodies

LPS 0111:B4 (L2630) and Doxycycline (D3447) were obtained from Sigma-Aldrich. Subcellular protein fractionation kit (78840) was obtained from Thermo Fisher. ATP (tlrl-atpl) was obtained from InvivoGen. StrataClean resin (400714) and chemical competent cells (200315) were obtained from Agilent. Bortezomib (B125789) and Carfilzomib (C127870) were obtained from Aladdin. PYR-41 (P798006) and Pevonedistat (MLN4924, P872287) were obtained from MACKLIN.

For FACS analysis, Annexin V-FITC/PI apoptosis kit (KGA1102) was obtained from KeyGEN BioTECH. For ELISA, mouse IL-1β kit (EM0109), mouse IL-18 kit (EM1158), mouse TNFα kit (EM0183), and mouse HMGB1 kit (EM0382) were obtained from Finetest. For immunoblotting, the Human Reactive Cell Death and Autophagy Antibody Sampler Kit (#42867, 1:1000), the Mouse Reactive Pyroptosis Antibody Sampler Kit (#98303, 1:1000), TRAF1 (#4715, 1:1000), OTUB1 (#3783, 1:1000), and LSD1 (#2184, 1:1000) were purchased from Cell Signaling Technology. Anti-Flag (F1804, 1 µg/mL) and anti-HA (H6908, 1:1000) were obtained from Sigma-Aldrich. Anti-β-Actin (66009-1-Ig, 1:1000) and anti-GST (10000-0-AP, 1:1000) were purchased from Proteintech. Anti-Na/K-ATPase α1 (PTM-5533, 1:1000) was purchased from PTO BIO.

### Stable cell lines

Lentivirus was produced in HEK293 cells by transfection of the lentiviral vector with psPAX2 (Addgene) and pMD2.G (Addgene). Lentiviral supernatants were collected, filtered through 0.45-μm filters, and used to transduce HEK293 cells. Polybrene infection/transfection reagent (Millipore, 10 μg/mL) was added to increase the efficiency of lentiviral infection. After two days of transduction, puromycin (Sigma, 2 μg/mL) was added to select the transduced cells. Empty lentiviral vectors were used to generate control cells. The expression of relevant genes in stable cell lines was verified by immunoblot.

### Immunoblots and immunoprecipitation

Cells were lysed in EBC buffer (50 mM Tris pH 7.5, 120 mM NaCl, 0.5% NP- 40) supplemented with protease inhibitors (A32953, Thermo Fisher) and phosphatase inhibitors (B15002, Bimake). The protein concentrations of lysates were measured using the Beckman Coulter DU-800 spectro-photometer and the Bio-Rad protein assay reagent. The same amounts of whole cell lysates were resolved by SDS-PAGE and immunoblotted with the indicated antibodies. For immunoprecipitation, cell lysates containing 1 mg of total proteins were incubated with anti-Flag-agarose Beads (A2220, Sigma) or anti-HA-agarose Beads (A2095, Sigma) for 4 hours at 4 °C. Precipitates were washed three times with EBC buffer and resolved by SDS-PAGE followed by immunoblot analysis with the indicated antibodies.

### GST pulldown

Cells were lysed in EBC buffer supplemented with protease inhibitors and phosphatase inhibitors. Cell lysates containing 1 mg of total proteins were incubated with 50 μL of GSH-agarose Beads (Beyotime, P2251) for 4 hours at 4 °C. Precipitates were washed thrice with EBC buffer and resolved by SDS-PAGE followed by immunoblot analysis with the indicated antibodies.

### NI/NTA pulldown

To purify His-Ub proteins, cell lysates were precipitated with nickel-nitrilotriacetic acid (Ni-NTA) agarose beads, resuspend 10 × 10^6^ cells in 1 mL of Buffer A (6 M guanidine-HCL, 0.1 M Na2HPO4, 0.1 M NaH2PO4, 10 mM imidazole, pH 8.0). Cell suspension was sonicated to disrupt viscous DNA to prevent it from interfering with the pull-down experiment, and then centrifuged C at 13,000 rpm for 5 minutes to discard cell debris. 50 μL of equilibrated (50%) Ni-NTA-agarose beads (QIAGEN, 30210) were added and incubatedfor 3 hours at room temperature. To remove unbound proteins, the beads were washed once with Buffer A and thrice with Buffer TI (Tris 25 mM, 20 mM imidazole, pH 6.8). Precipitates were resolved by SDS-PAGE, followed by immunoblot analysis with indicated antibodies.

### Protein enrichment from the culture medium or peritoneal fluid

To enrich the proteins in the culture medium, 1 mL of culture medium was centrifuged at 14,000 × *g* for 10 minutes at 4 °C to remove cellular debris. 10 μL of StrataClean resin (400714, Agilent) was then added for 1 hour incubation on a rotator at 4 °C. The supernatants were removed by centrifugation. The resin was harvested and suspended in 50 μL of 2 × loading buffer for immunoblotting. In the septic mouse model, the proteins in flushed peritoneal fluid were also enriched in the same way.

### Size exclusion chromatography (SEC)

SEC was performed using an ÄKTA Purifier system (GE Healthcare, Buckinghamshire, England). A HiLoad 16/600 Superdex 200 column (GE Healthcare) was equilibrated with cell lysis buffer. The column was calibrated using a gel filtration calibration kit (GE Healthcare). Each standard protein was dissolved in cell lysis buffer and chromatographed on the column separately. The filtered protein samples were then fractionated on the column (1.0 mL/min, 2 mL/fraction). For immunoblot analysis, proteins in the fractionated eluent were enriched with 10 μL of StrataClean resin and resuspended in 30 μL of 2 × loading buffer.

### Cytotoxicity assay and cell viability assay

Cell death and cell viability were assessed using Non-Radioactive Cytotoxicity Assay Kit (G1780, Promega) and CellTiter-Glo Luminescent Cell Viability Assay Kit (G7571, Promega), respectively. Briefly, 5 × 10^3^ cells were cultured in 96-well plates with an opaque wall. At the desired time points, cell death was determined by titrating the amount of lactate dehydrogenase released into the culture medium, and cell viability was determined by the ATP levels within cells, according to the manufacturer’s instructions.

### Mouse studies

All procedures were conducted in accordance with institutional animal care and use guidelines and were approved by the Zhengzhou University Animal Care Committee (ZZU-LAC20231229[04]). Female wild-type C57BL/6 and NSG mice (6–8 weeks old) were purchased from Vital River Laboratories. All mice were housed in the Zhengzhou University Animal Facility.

For the xenograft experiment, before inoculation, cell viability was determined using the trypan blue exclusion test (minimum of 98% cell viability). 1.0 × 10^6^ cells were subcutaneously injected into the right flank of mice. Tumor growth was monitored every other day. Tumor volume (mm^3^) is calculated via the “(W × W × L) / 2” formula, where L is the longest diameter and W is the shortest diameter. Necropsy and tumor collection were performed at the end of the tumor size recording.

For the septic model, mice were pretreated with PYR-41 (10 mg/kg, i.p.) on day 14 and the following every other day. On day 0, LPS (5 mg/kg, i.p.) was injected to induce sepsis. The sera were harvested for ELISA assay at 6 hours post-LPS.

### Flow cytometry

FACS analyses were performed according to the manufacturer’s guidelines. Briefly, cells were digested using trypsin without EDTA to get cell suspension. Then, cells were washed with PBS twice and resuspended in 500 μL of Binding Buffer to form a single-cell suspension. After adding 5 μL of Annexin V-FITC and 5 μL of Propidium Iodide, cells were incubated at room temperature for 5 minutes before conducting flow cytometry analysis.

### Immunofluorescence staining and fluorescent microscopy

For Immunofluorescence staining, the dish with special glass bottom was used. Cells were fixed with 100% methanol (chilled at −20 °C) at room temperature (RT) for 5 minutes. To block nonspecific binding of antibodies, cells were incubated with 1% BSA, 22.52 mg/mL glycine in PBST ( 0.1% Tween 20 in PBS) for 30 minutes (alternatively we used 10% serum from the species in which the secondary antibody were prepared). For immunostaining, cells were incubated with the diluted antibody in 1% BSA in PBST in a humidified chamber for 1 hour at RT or overnight at 4 °C and then incubated with the secondary antibody in 1% BSA for 1 hour at RT in the dark. Finally, cells were counter-stained with 0.1–1 µg/mL Hoechst or DAPI for 1 minute, mounted on the slides and subjected to microscopic analysis. Note: Between steps, cells were washed with PBS for 3 times, 5 minutes per wash.

For fluorescent microscopy of live cells, PI (10 µg/mL) or DAPI (1 µg/mL) was added to culture medium. 10 minutes later, cells were imaged under fluorescent microscope.

### Statistical analysis

Student’s *t*-test was used to determine the differences between the two groups. Differences between tumor growth curves were compared by calculating the area-under-curve (AUC) values for each sample and then comparing different groups using Student’s *t*-test. The results are presented as the mean and standard error of the mean (SEM). Statistical significance was assigned to *P* < 0.05. Tumor-free survival analysis and Kaplan-Meier analysis were performed using GraphPad Prism 5.04 for Windows.

## Supplementary information

GD-NT interactome for for publication

GD-Ub SM 20241229

Uncropped original western blot 20241124

## Data Availability

All relevant data are available in the Source Data (for Figs. 1–6) or supplementary information associated with this study.
