# Supplementary material for: Ubiquitination of gasdermin D N-terminal domain directs its membrane translocation and pore formation during pyroptosis
Source: Cell Death Dis. 2025 Mar 17;16(1):181. doi: 10.1038/s41419-025-07475-6 (PMC11914233; doi:10.1038/s41419-025-07475-6)
Supplement: Supplementary file 2 — GD-Ub SM 20241229 [file 41419_2025_7475_MOESM2_ESM.docx]

Supplementary Materials for

**Ubiquitination of gasdermin D N-terminal domain directs its membrane translocation and pore formation during pyroptosis**

Xiufeng Chu^1,2†^*, Ting Zhang^1†^, Ihtisham Bukhari^2†^, Mei Hu^1^, Jixuan Xu^3^, Yamin Xing^2^, Xinfeng Liang^2^, Zisen Zhang^1^, Pengyuan Zheng^2^

^1^Department of Oncology, The Fifth Affiliated Hospital of Zhengzhou University, Zhengzhou, China.

^2^Marshall B. J. Medical Research Center, The Fifth Affiliated Hospital of Zhengzhou University, Zhengzhou, China.

^3^Department of Gastrointestinal & Thyroid Surgery, The Fifth Affiliated Hospital of Zhengzhou University, Zhengzhou, China.

^†^ These authors contributed equally to the work

* Corresponding author: Xiufeng Chu: xchu@zzu.edu.cn

**This file includes:**

**Extended Fig. 1 to 5 and Extended Table 1**

**Extended Fig. 1 | The pyroptosis signaling pathway and NF-κB signaling pathway.**

A. In the classical pyroptosis pathway, intracellular pattern recognition receptors PRRs (NLRP3, NLRC4, NLRP1B, etc.) recognize pathogenic stimuli and bind to pro caspase-1 through the adapter protein ASC, forming inflammasomes to generate active caspase-1. In the atypical pyroptosis pathway, intracellular LPS directly binds and activates pro caspase-11 to generate active caspase-11. After activation of inflammatory caspase, pro-IL-1 β and pro-IL-18 are cleaved into active IL-1 β and IL-18. The GSDMD portion connecting the N-end and C-end is rapidly cut to remove the inhibitory effect of the C-end on the N-end. Then, the N-terminal of GSDMD connects to phosphatidylinositol (PI) on the cell membrane, producing oligomerization and forming a “gasdermin channel”. Ion movement through this channel destroys the osmotic balance, leading to cell swelling, membrane dissolution, cell content release, and inflammatory response. Full English name of professional terms: PRRs: Pattern recognition receptors. NLRP3: Nod-like receptor (NLR) family pyrin domain containing 3. NLRC4: NLR family caspase activation and recruitment domain (CARD) containing 4. NLRP1B: NLR family pyrin domain -containing 1B. ASC: apoptosis-associated speck-like protein contain a CARD.

B. The relationship between the NF- κB signaling pathway and cell death. TNFR received NF- κ After the B signal, different complexes are formed within the cells, including Complex I, Complex Iia, and Complex Iib, which respectively regulate the expression of inflammatory genes and cell survival, apoptosis, and necrosis. IAP induced RIPK1 ubiquitination, leading to NF- κ Activation of B and subsequent inflammatory cascade, as well as phosphorylation of RIPK1 at the Ser320 site. Deubiquitinases such as CYLD and A20 can inhibit complex I, thereby promoting the interaction between RIPK1 and caspase-8, as well as the death-inducing complex IIa pathway. The interaction with RIPK3 is necessary for complex IIb signal transduction and necroptosis. Caspase-8 activity can inhibit necrotic apoptosis by cleaving RIPK1 and RIPK3.

**Extended Fig. 2 | Pyroptosis cell model 293-tetO-GD-NT is set up to study the regulation mechanisms of pyroptosis.**

A. Tetracycline on (TetOn) system uses tetracycline (or one of its analogs like doxycycline) as a regulator of gene expression. A tetracycline-dependent promoter is created by placing a TRE upstream of a minimal promoter. TRE is seven repeats of the tetracycline operator (tetO) sequence and is recognized by a **r**everse **t**etracycline-controlled **t**rans**a**ctivator (rtTA). In the presence of tetracycline or one of its analogs like doxycycline (Dox), rtTA binds to the TRE and tetracycline, permitting transcription. To create 293-tetO-GD-NT cells, HEK293 cells were transduced with lentivirus to express Dox-inducible GD-NT.

B. Immunoblot (IB) analysis of 293-tetO-GD-NT cells. Cells were treated with Dox (2 µg/ml) and harvested at different time points to obtain whole cell lysate (WCL). IB analysis of WCL was performed with various antibodies. β-actin served as internal control.

C. Similar to (B), except that the cells were subjected to ATP-based Cell Viability Assay.

D. Similar to (B), except that the cells were subjected to LDH-based Cytotoxicity Assay.

E. Similar to (B), except that the morphological changes were observed using phase-contrast imaging (lower panel). Dying cells were stained with DAPI for fluorescent microscope imaging (upper panel).

F. Similar to (B), except that cell death was analyzed by flow cytometry using apoptosis-Annexin V/PI staining kit.

**Extended Fig. 3 | PTMs contribute to the regulation of gasdermin activity.**

A. Succination prevents GSDMD activation. The intermediate of the tricarboxylic acid cycle, fumarate, and its dimethyl DMF react with the cysteine residue of GSDMD to form S - (2-butyryl) - cysteine, also known as “ Succination ”. Succination prevents the interaction between GSDMD and caspase-11, making the former impossible to be processed to produce the active fragment GD-NT with the ability to kill cells.

B. Palmitoylation of GD-NT enhances its pore-forming activity. S-palmitoylation of GSDMD at Cys191/192 (human/mouse) catalyzed by palmitoyl acyltransferases ZDHHC5 and ZDHHC9 directly mediates membrane translocation of GSDMD-NT but not full-length GSDMD.

C. Phosphorylation of GD-NT suppresses its pore-forming activity. AMP-activated protein kinase (AMPK) catalyzes the phosphorylation of GD-NT at Ser45/46 (human/mouse) and negatively regulates the cytolytic activity of GD-NT.

**Extended Fig. 4 | PTMs contribute to the regulation of gasdermin activity.**

A. Pathogenic ubiquitination inhibits GSDMD-mediated pyroptosis in epithelial cells. GSDMD executes pyroptosis to eliminate the replicative niche of intracellular pathogens. However, Shigella ubiquitin ligase IpaH7.8 specifically ubiquitinates human, but not mouse, GSDMD and targets it for proteasomal degradation.

B. Pathogenic ubiquitination of GSDMB inhibits the bactericidal function of NK cells. Granzyme A secreted by NK cells can enter epithelial cells infected with intracellular bacteria and activate GSDMB, which recognizes and binds phospholipids on bacterial membranes, causing intracellular bacteria to undergo pyroptosis. As one of the mechanisms that resist host immune killing, the ubiquitin ligase (E3) IpaH7.8 secreted by invasive Shigella flexneri causes GSDMB ubiquitination and subsequent proteasomal degradation, thereby rendering the Granzyme A-GSDMB pyroptosis system ineffective.

**Extended Fig. 5 | PTMs contribute to the regulation of gasdermin activity.**

A. Pyroposis-related pathological conditions and Clinical translation studies related to PTM. Among them, the diseases closely related to pyroptosis include sepsis, CAPS, and MAS. The diseases that are moderately associated with pyroptosis include type 2 diabetes, obesity, myocarditis, atherosclerotic diseases, and gouty arthritis. Clinical translational studies of PTMs and GSDMD include succination, palmitoylation, phosphorylation, and the formation of disulfide bonds. The drugs targeting these PTMs are DMF, PMB, metformin, and disulfiram, respectively.

Abbreviations: DMF, dimethyl fumarate; PMB, palmostatin B; CAPS, Cryopyrin-Associated Periodic Syndromes; MAS, Macrophage Activation Syndrome.

**A**

| **Inflammasome Signaling-related E3/DUBs** | | | |
| --- | --- | --- | --- |
| Class | E3/DUB name | Target | Outcome for  pyroptosis |
| E3 | Pellino2, TRAF6, TRIM33 | NLRP3-K63-Ub | activation |
|  | TRIM33 | DHX33 | activation |
|  | TRAF3, LUBAC | ASC | activation |
|  | Cullin1, ARIH2 | NLRP3-K48-Ub | inactivation |
|  | TRIM31, FBXL2, PARKIN | NLRP3-K48-Ub | inactivation |
|  | MARCH7 | NLRP3-K48-Ub | inactivation |
|  | TRAF6 | ASC-K63-Ub | inactivation |
| DUB | USP7, USP47, UCHL5, BRCC3/ABRO1 | NLRP3-K48-Ub | activation |
|  | USP50 | ASC | activation |
|  | A20 | NLRP3-K63-Ub | inactivation |

**B**

| **NF-κB Signaling-related E3/DUBs** | | | |
| --- | --- | --- | --- |
| Class | E3/DUB name | Target | Outcome for  NF-κB Signaling |
| E3 | TRAF2/5-TRADD-cIAP1/2 | RIPK1-K63-Ub | activation |
|  | LUBAC | NEMO-Lin-Ub | activation |
|  | Pellino-1 (PELI1) | IRAK1/4 | activation |
|  | TRAF6 | NEMO | activation |
|  | β-TrCP | I𝜅B-K48-Ub  p100-K48-Ub | activation |
|  | TRAF2-TRAF3-cIAP1/2 | NIK-K48-Ib | inactivation |
|  | XIAP | RIPK1-K63-Ub  RIPK2-K63-Ub | Inactivation |
| DUB | CYLD | RIPK1-K63-Ub  NEMO-Lin-Ub | inactivation |
|  | OTULIN | NEMO | inactivation |
|  | DUBA | TRAF3 | inactivation |
|  | A20 | RIPK1-K63-Ub  NEMO-Lin-Ub | inactivation |

**Extended table 1 | The contribution of ubiquitination system in pyroptosis.**

A. Regulation of NLRP3 inflammasome by the ubiquitin system. The ubiquitin system contributes to both negative (i) and positive (ii) regulation of NLRP3. (i) E3 ligases such as FBXL2, PARKIN, TRIM31, or MARCH7 have been identified as negative regulators of the inflammasome by controlling its protein levels by either proteasome or autophagy pathways. Upon sensing inflammasome-priming signals (signal 1), ARIH2 and Cullin1 bind and ubiquitinate NLRP3 to maintain it inactive, preventing its assembly and inflammasome activation. Upon sensing activating signals (signal 2), they dissociate from NLRP3, reducing its ubiquitination and allowing for the formation of an active inflammasome. The deubiquitinase A20 also contributes to the negative regulation of the NLRP3 inflammasome. (ii). On the contrary, ubiquitination of NLRP3 by Pellino2, TRAF6, and TRIM33 upon signal 1 poises the NLRP3 into an active estate that facilitates its activation by signal 2. Upon sensing these activating signals, deubiquitinases such as BRCC3/ABRO1, USP7, USP47, or UCHL5 contribute to inflammasome activation, although the exact mechanisms by which they do this are not completely understood.

B. Regulation of NF-κB Signaling pathway by the ubiquitin system.
