# Supplementary material for: Ubiquitination of gasdermin D N-terminal domain directs its membrane translocation and pore formation during pyroptosis
Source: Cell Death Dis. 2025 Mar 17;16(1):181. doi: 10.1038/s41419-025-07475-6 (PMC11914233; doi:10.1038/s41419-025-07475-6)
Supplement: Supplementary file 3 — Uncropped original western blot 20241124 [file 41419_2025_7475_MOESM3_ESM.pdf]

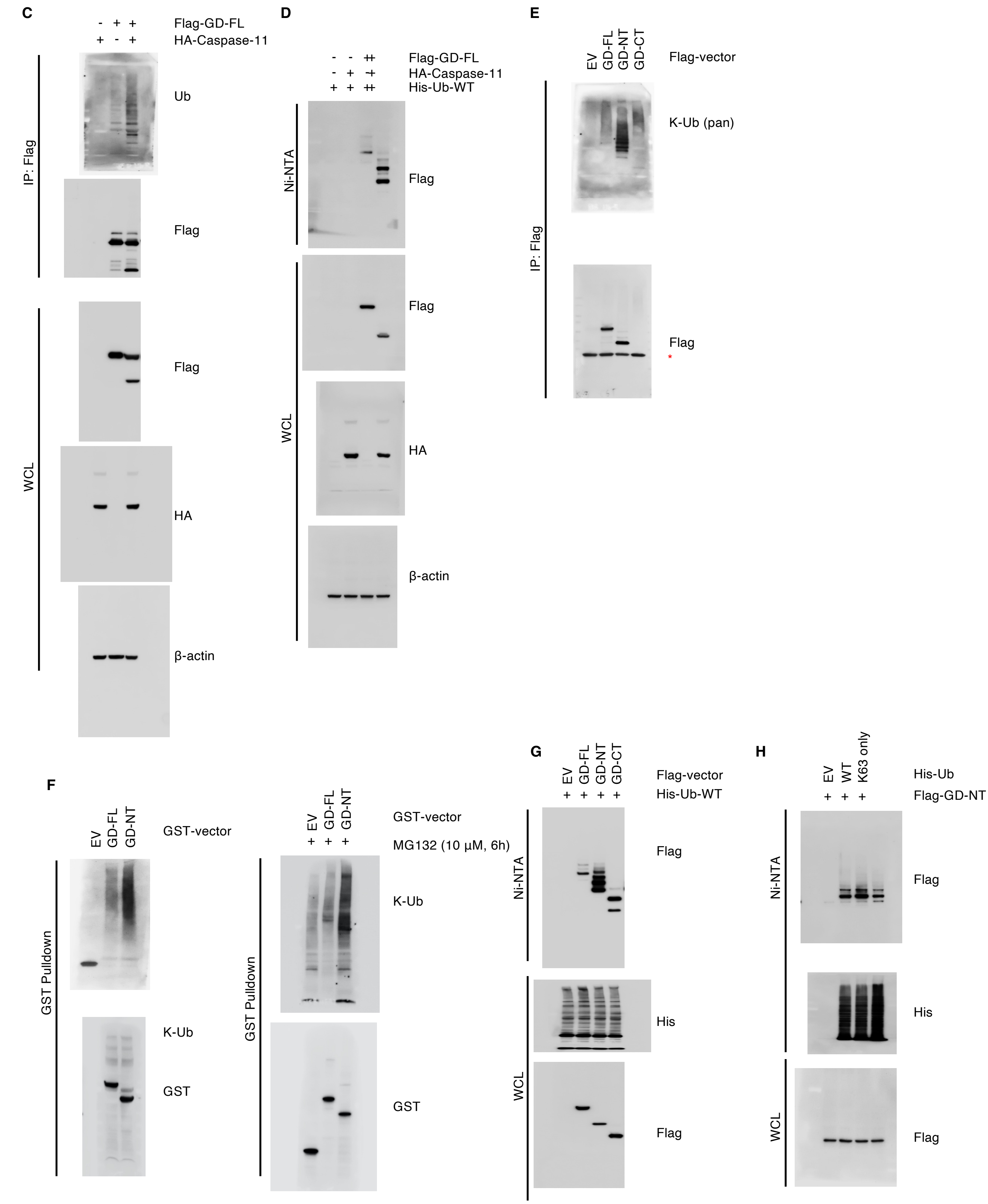

Fig. 1 | GD-NT, but not GD-FL, possesses massive K63-linked polyubiquitin.

H

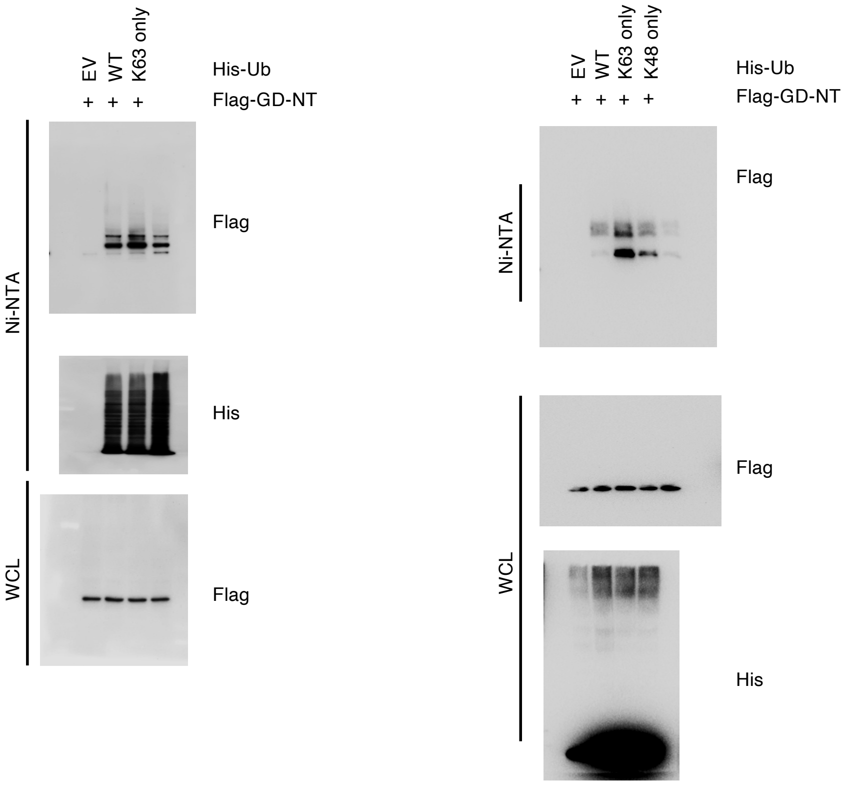

Fig. 1 | GD-NT, but not GD-FL, possesses massive K63-linked polyubiquitin.

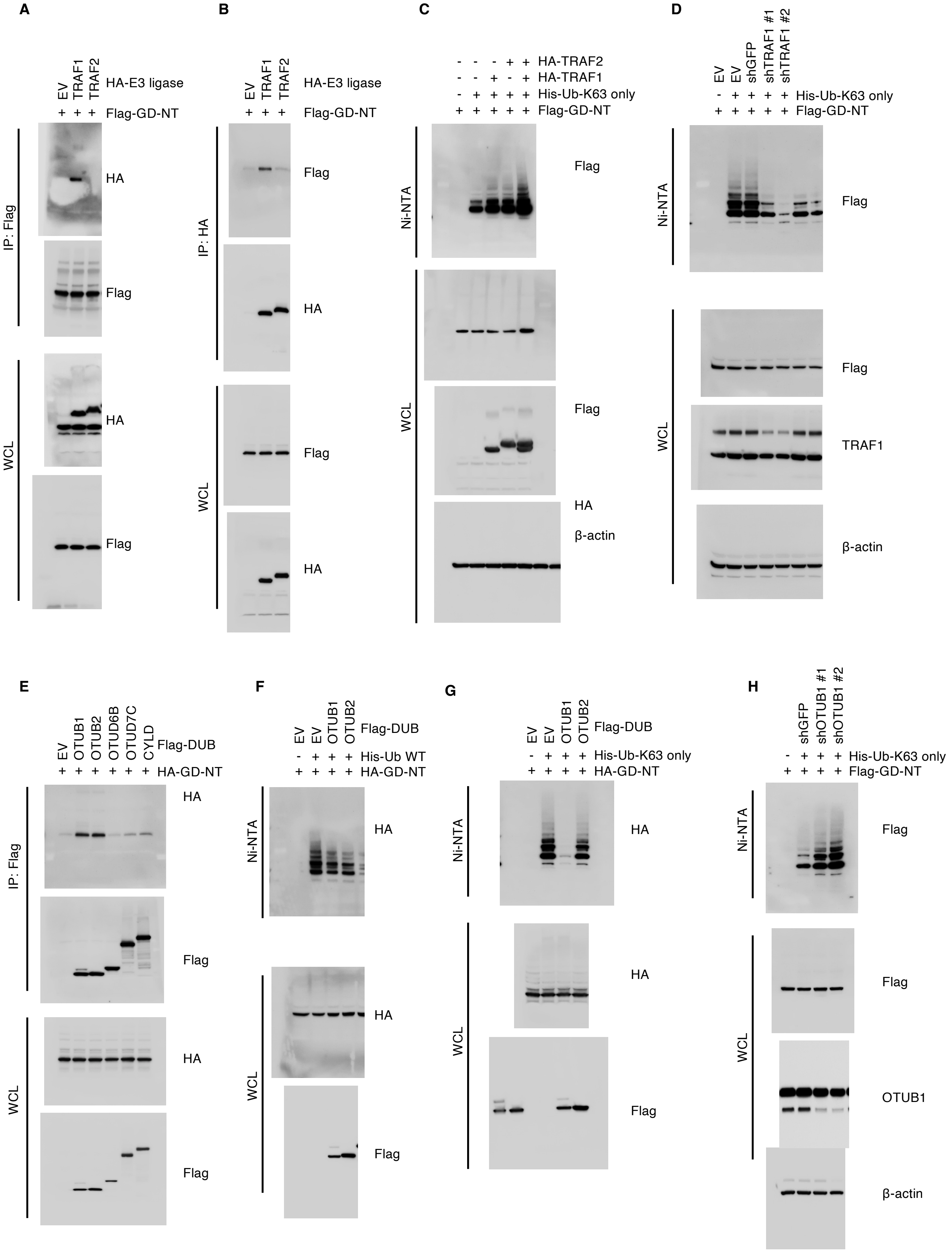

Fig. 2 | The K63-linked polyubiquitin of GD-NT is regulated by TRAF1-OTUB1 axis.

K

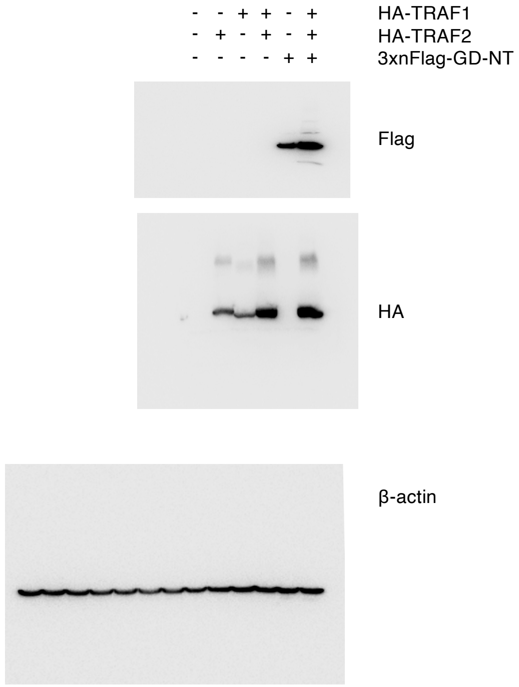

Fig. 2 | The K63-linked polyubiquitin of GD-NT is regulated by TRAF1-OTUB1 axis.

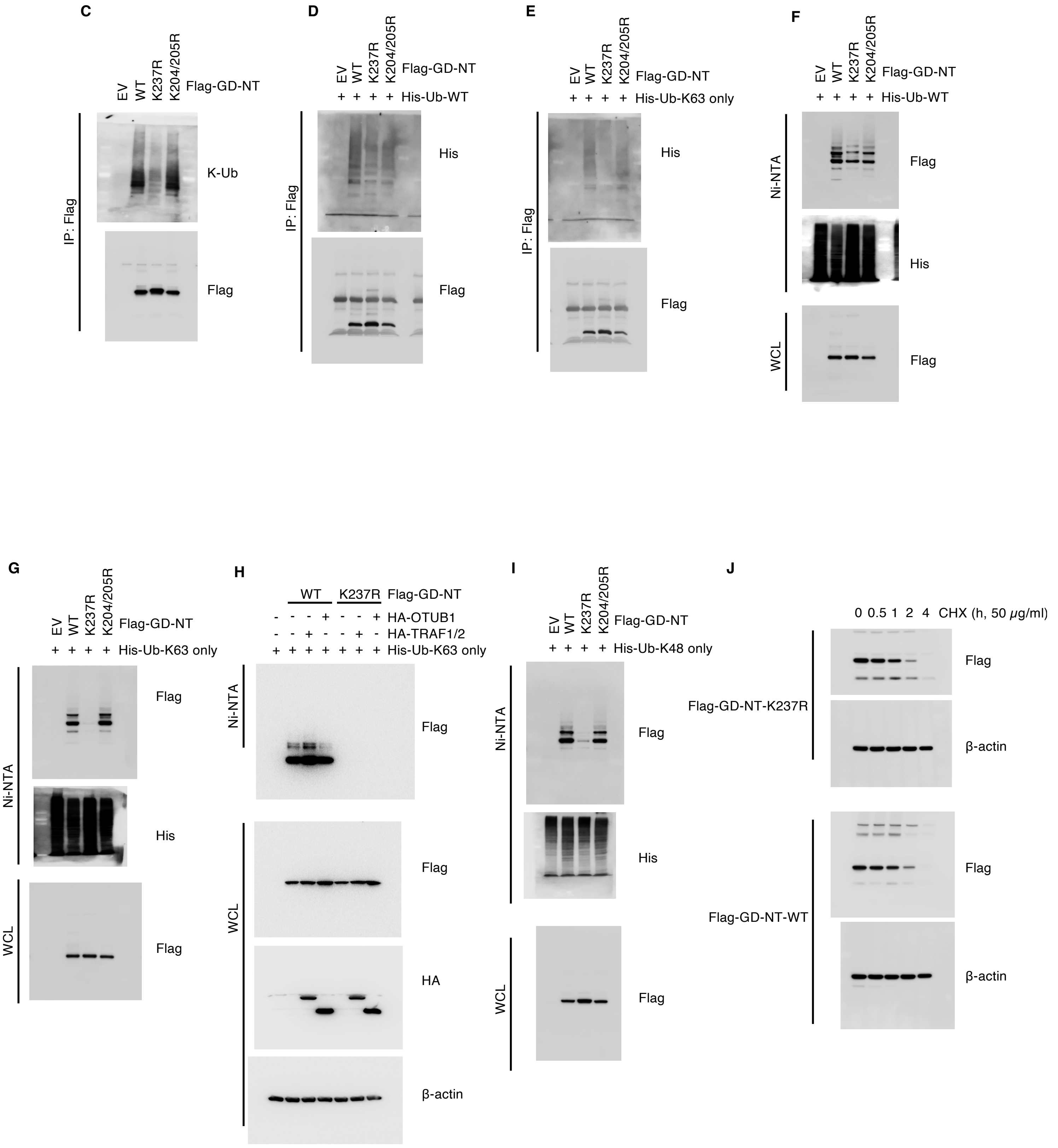

Fig. 3 | The amino acid Lys237 of GD-NT possess K63-type ubiquitination.

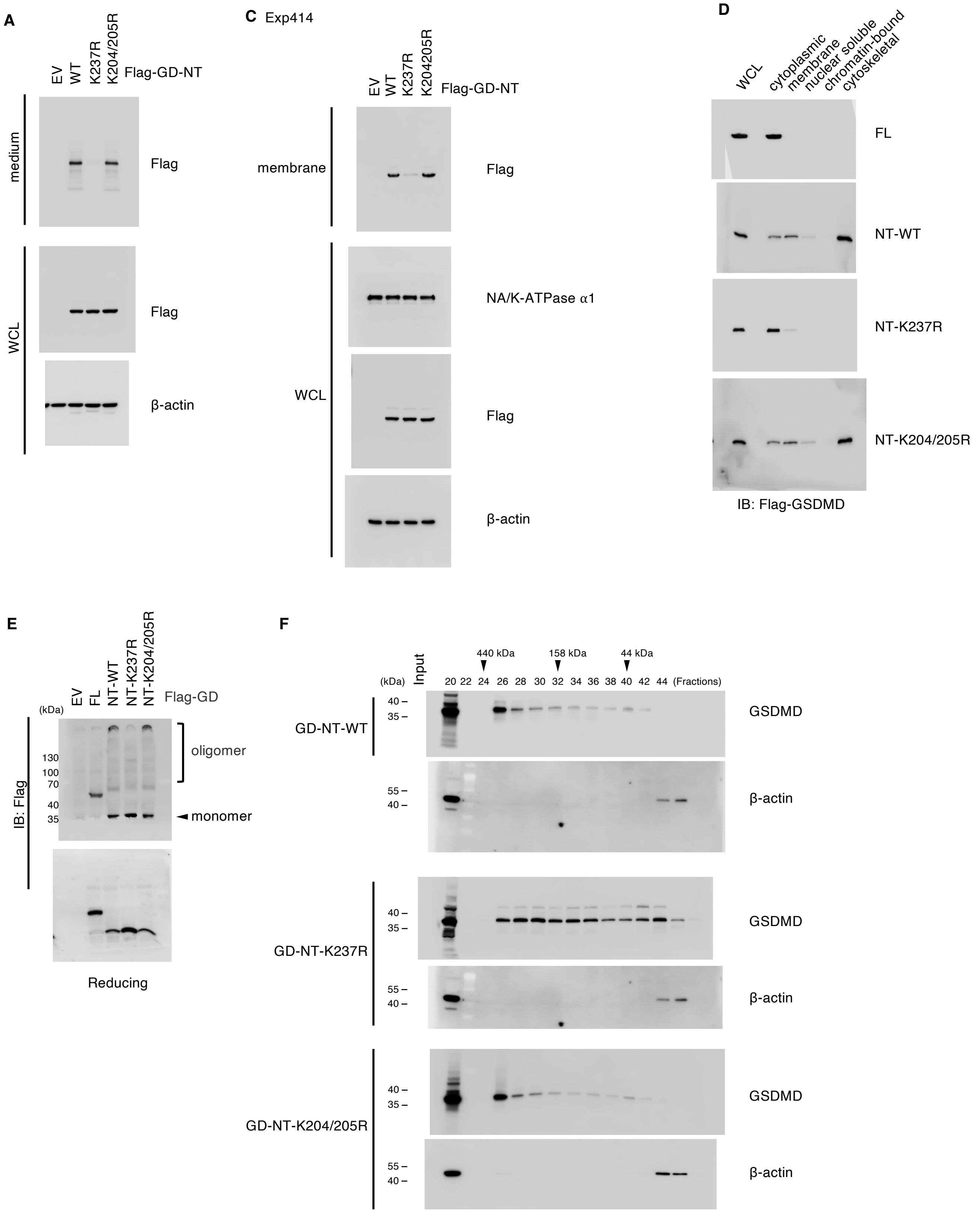

Fig. 4 | Ubiquitination on Lys237 of GD-NT affects its localization and oligomerization.

A

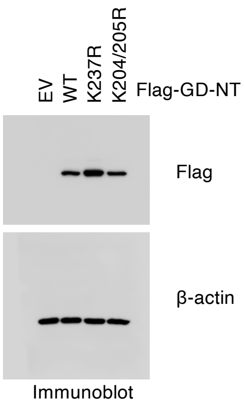

Fig. 5 | Ubiquitinated GD-NT loses the ability to mediate pyroptosis in vivo and in vitro.

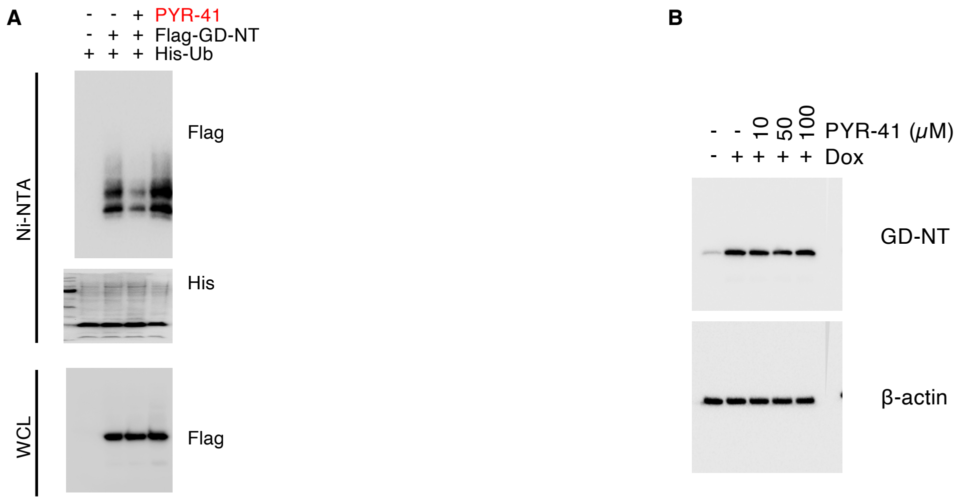

Fig. 6 | PYR-41 suppressed GD-NT ubiquitination and reduced GD-NT-mediated pyroptosis in vivo and in vitro.

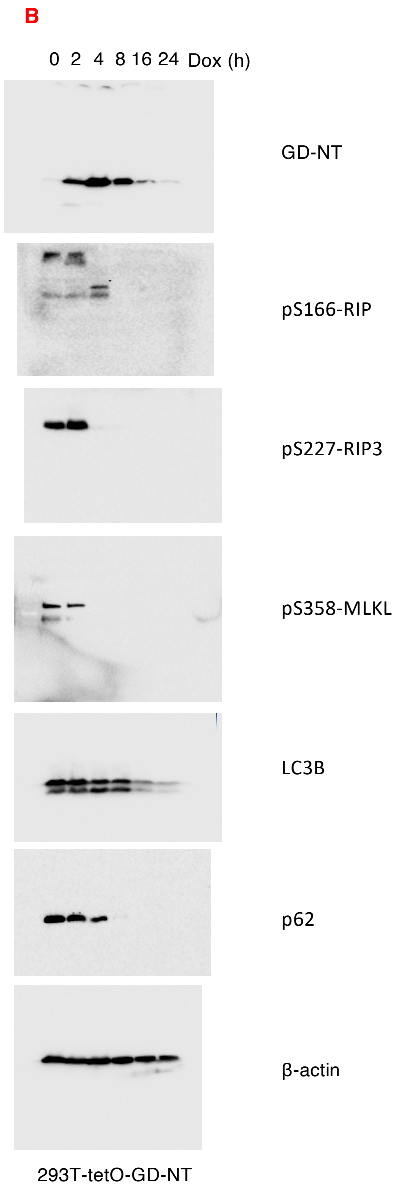

Extended Fig. 2 | Pyroptosis cell model 293-tetO-GD-NT is set up to study the regulation mechanisms of pyroptosis.
